# Kinesin-1 captures RNA cargo in its adaptable coils

**DOI:** 10.1101/gad.348691.121

**Published:** 2021-07-01

**Authors:** Jessica A. Cross, Derek. N. Woolfson, Mark P. Dodding

**Affiliations:** 1 School of Biochemistry, Faculty of Life Sciences, University of Bristol, Bristol BS8 1TD, United Kingdom;; 2 School of Chemistry, Faculty of Life Sciences, University of Bristol, Bristol BS8 1TS, United Kingdom;; 3 Bristol BioDesign Institute, University of Bristol, Bristol BS8 1TQ, United Kingdom

**Keywords:** kinesin, kinesin adaptor, kinesin–atypical tropomyosin complex, mRNA transport, oskar mRNA

## Abstract

The prototypic and ubiquitous microtubule motor, kinesin-1, uses a variety of adaptor proteins to facilitate the selective transport of diverse cargo within the cell. These cargo adaptors bind to the motor complex through interactions with the kinesin light or heavy chains (KLCs or KHCs). In this issue of *Genes & Development*, Dimitrova-Paternoga et al. (pp. 976–991) present the first structural characterization of a KHC–cargo adaptor interface. They describe an antiparallel heterotrimeric coiled-coil complex between the carboxy tail of KHC and Tm1-I/C (*a*Tm1), the atypical tropomyosin that is important for *oskar* mRNA transport in *Drosophila* oocytes. This interaction enhances direct binding between KHC and RNA. Their findings demonstrate the structural plasticity of the KHC tail as a platform for protein–protein interactions and reveal how a cargo adaptor protein can modify a motor–RNA interface to promote transport.

## Abstract (precis)

This Outlook discusses the findings by Dimitrova-Paternoga et al. revealing an antiparallel trimeric coiled-coil complex between the C-terminal tail region of KHC and *a*Tm1 that enhances direct binding between KHC and RNA.

Kinesin-1 family microtubule motor proteins play a key role in intracellular transport in most cell types. They can engage diverse cargos including membrane-bound organelles (MBOs), proteins, and RNA, as well as many viruses and slide microtubules to control the organization of the microtubule network itself (Verhey and Hammond 2009; Lu and Gelfand 2017; Cross and Dodding 2019). To meet these complex functional requirements, kinesin-1 must specifically, and selectively, recognize cargos, and those interactions must be regulated. This is achieved in part through the binding of cargo adaptor proteins. These can be loosely defined as molecules that interact directly or indirectly with cargo and directly with a motor protein complex. As such, they define a key motor–cargo interface acting as bridges and regulatory hubs that control motor recruitment and activation. Recent structural studies have begun to show how these adaptors support selective MBO recognition via the KLCs (Cross and Dodding 2019), but much less is understood about how they enable recognition, recruitment, and transport of RNA.

Dimitrova-Paternoga et al. (2021) explore the role of the atypical tropomyosin, Tm1-I/C (*a*Tm1), that is important for *oskar* mRNA localization to the posterior pole of the *Drosophila* oocyte. They solve X-ray crystal structures of an antiparallel homodimeric *a*Tm1 coiled coil in addition to a heterotrimeric complex consisting of two parallel KHCs and one antiparallel *a*Tm1 chain. The structure of the trimeric complex is validated by mutagenesis and biochemical and in vivo RNA transport assays. The KHC–*a*Tm1 complex is shown to bind RNA with higher affinity than KHC only, suggesting a new mechanism by which kinesin–cargo (RNA) transport can be modulated through stabilization by a KHC cargo adaptor. This is likely due to the positively charged binding surface formed in the trimeric KHC–*a*Tm1 complex and possible stabilization of an extended helical region of the KHC tail. To our knowledge, this is the first structural characterization of a direct cargo adaptor–KHC interface, giving important new insight into the mechanism of RNA recognition.

The region of KHC shown to bind *a*Tm1 immediately follows the sequences of KHC that are responsible for binding the KLCs (Fig. 1A,B; Diefenbach et al. 1998). This region also interacts with several other cargo adaptors and contains the ATP-independent microtubule binding site required for microtubule–microtubule sliding (Verhey and Hammond 2009; Lu and Gelfand 2017; Sanger et al. 2017). It will be important to explore whether and how the mode of binding *a*Tm1 extends to other adaptors and whether this influences motor–microtubule interactions. It is notable that RNA interaction with the KHC–*a*Tm1 complex is most likely enhanced due to presentation of an expanded positively charged binding surface; it seems possible that a related mechanism could also promote binding to the acidic tubulin C-terminal tails.

**Figure 1.**
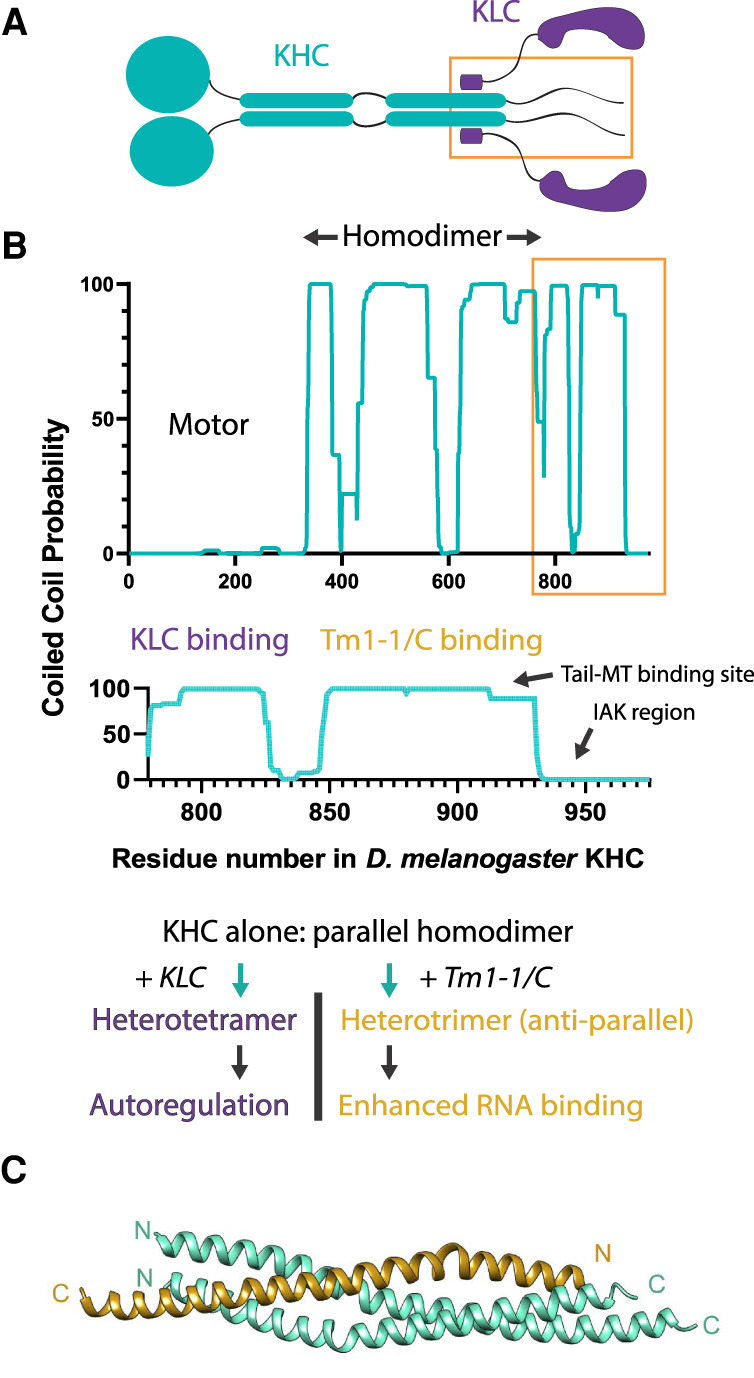
Structural and functional plasticity of the KHC coiled coils. (*A*) Schematic of the kinesin-1 heterotetramer. (Cyan) KHC, (purple) KLC. The orange boxed region highlights the KHC tail. (*B*, *top*) Marcoil (https://toolkit.tuebingen.mpg.de/tools/marcoil) coiled-coil prediction for KHC; tail region is boxed orange. (*Middle*) Detailed coiled-coil prediction for the KHC tail that contains the binding sites for KLC and Tm1-I/C. (*Bottom*) Potential structural plasticity and associated function in the KHC coils. (*C*) Crystal structure of the KHC–KHC–Tm1-I/C complex (PDB: 7BJS) from Dimitrova-Paternoga et al. (2021) (Cyan) KHCs, (brown) Tm1-I/C.

Previous studies have suggested that KLC is also important for Staufen/*oskar* localization (Lu et al. 2018) through interplay with another tetratricopeptide repeat protein, PAT1 (Loiseau et al. 2010). In addition to their role in cargo recognition, KLCs are also important for mediating kinesin-1 autoinhibition, in a manner dependent on sequences containing their KHC binding heptad repeats (Verhey et al. 1998). Therefore, if some cargos are transported in a truly KLC-independent mechanism (i.e., KLC is not a component of the complex), this may suggest a secondary role for KHC tail-binding cargo adaptors such as *a*Tm1 in motor regulation, akin to that described for the KLCs. This seems plausible given that the *a*Tm1 binding site is sandwiched between the KLC binding site and the IAK region that interacts with the motor domains to mediate autoinhibition (Fig. 1B). However, a recent study in mammalian cells also mapping RNA/cargo adaptor binding determinants showed that SFPQ-RNA granules are transported by kinesin-1 tetramers comprised of KIF5A (a neuronal mammalian KHC paralog, also implicating the KHC tail in binding) and KLC1, suggesting that the picture may be quite complex and could differ between RNA cargo and/or species (Fukuda et al. 2020).

Coiled coils most commonly form dimers, trimers, and tetramers in nature, with control over oligomeric state and orientation largely directed by patterns of isoleucine (Ile [I]), leucine (Leu [L]) in the core (heptad *a* and *d* positions), and salt bridges formed by adjacent residues (*g* and *e* positions). As the investigators noted, the finding that the *a*Tm1 constructs crystallize as antiparallel coiled coils is in itself interesting; the received wisdom is that tropomyosins form exclusively parallel coiled-coil dimers (Hitchcock-DeGregori and Barua 2017). Therefore, for these constructs to behave differently from the norm is worth reflection. First, the *a* and *d* sites that define the hydrophobic part of the helix–helix interface are predominantly aliphatic hydrophobic with few obvious features that might discriminate between different coiled-coil structures. In addition, we note that the acidic and basic side chains at the *g* and *e* positions (Fig. 4A in Dimitrova-Paternoga et al. 2021) could possibly be better accommodated in a parallel arrangement. Thus, it is possible that these regions of the *a*Tm1 sequence are somewhat promiscuous or agnostic with regard to coiled-coil partner selection and orientation. In turn, this could contribute to its adaptability as the investigators elegantly and persuasively show. In contrast to *a*Tm1, the two KHCs retain a parallel interaction in the complex (Fig. 1C). Nonetheless, to form a trimeric hydrophobic core with the additional *a*Tm1 helix, the KHC coils must also show conformational flexibility to open up the interface. This must also be true of the adjacent KLC binding site, where the KHCs presumably undergo a transition from a homodimeric to heterotetrameric coiled-coil assembly to accommodate the KLC heptad repeats (Fig. 1B). It is not clear whether this interaction is parallel or antiparallel. It will be interesting to discover whether KLC heterotetramer and cargo adaptor heterotrimer states can occur simultaneously.

Together, these data form a picture of the KHC stalk as a dynamic and flexible platform for protein–protein interactions. This is in contrast to the rigid spacer-like properties with functions limited to oligomerization sometimes associated with coiled-coil domains. This could play an important role in the larger conformational changes associated with transition from a compact autoinhibited state to an extended active form of the motor, capable of motility on the cytoskeleton. Dimitrova-Paternoga et al. (2021) provide important molecular insight into how RNA cargos are recognized by this crucial molecular machine. Their findings suggest that it is now time for the often-neglected kinesin-1 coiled coils to move to the fore as we expand our understanding of regulation and its coupled, remarkably versatile, cargo selection mechanisms.
